# Assessing construct validity of the Grit-S in Chinese employees

**DOI:** 10.1371/journal.pone.0209319

**Published:** 2018-12-21

**Authors:** Chuxian Zhong, Meng-Cheng Wang, Yiyun Shou, Fen Ren, Xintong Zhang, Mingshu Li, Wendeng Yang

**Affiliations:** 1 Department of Psychology, Guangzhou University, Guangzhou, China; 2 The Center for Psychometrics and Latent Variable Modeling, Guangzhou University, Guangzhou, China; 3 The Key Laboratory for Juveniles Mental Health and Educational Neuroscience in Guangdong Province, Guangzhou University, Guangzhou, China; 4 The Australian National University, Canberra, Australia; 5 School of Education and Psychology, University of Jinan, Jinan, China; University of Hong Kong, HONG KONG

## Abstract

This research examined the psychometric properties and construct validity of the Short Grit Scale (Grit-S) in Chinese insurance employees (*N* = 2,363; 37% males; mean age = 35.14). Exploratory factor analysis and confirmatory factor analysis (CFA) were used to determine the factor structure of the Grit-S. The resulting model was tested by multi-group CFA for the factorial invariance of the Grit-S across genders and age groups. Results showed that the Grit-S could be best explained by a two-factor model containing consistency of interest (α = .70) and perseverance of effort (α = .75). The factor model was equivalent across genders and age groups. The scores of the Grit-S were significantly correlated with external criteria variables including mental wellbeing and job performance. Overall, our findings suggested that the Grit-S can be a promising assessment of the grit trait in Chinese employees.

## Introduction

Grit was initially proposed as the trait of strenuously sustaining ambition regardless of failure or adversity. It requires that an individual show persevering effort and sustaining consistent interest [1]. The concept is different from cognitive ability, but essential to success. Grit is found to be a valid construct in both adolescents and adults in various domains [1], such as academic [2] or professional [3].

To measure grit, Duckworth and colleagues (2007) [1] developed a 12-item scale based on the theory that grit entails components of (1) perseverance of effort and (2) consistency of interest. The two-factor structure of the scale was supported in an exploratory factor analysis (EFA) [1]. Duckworth and Quinn revised this scale and reduced the scale to eight items, naming it Grit-S [4]. The Grit-S has a hierarchical model structure, with two factors of the Grit–S loaded on a second-order latent factor called grit [4].

A recent meta-analysis [5] pointed out that the hierarchical model structure of the Grit-S is problematic [5] as the second-order factor has only two first-order factors as indicators. The effects of the second-order factor on the first-order factors may not be identifiable without imposing additional constraints, such as constraining the factor loadings of the two first-order factors equally onto the second-order factor. Such a hierarchical model becomes no different from a simple single-factor structure with two-correlated first-order factors.

Early examinations of the psychometric properties of the Grit-S used primarily American samples, most of whom were undergraduate students [4, 6, 7]. The findings on the validity and reliability of the Grit-S in samples from other cultural and language backgrounds were mixed. Studies that provided some support for the Grit-S covered samples which included Chinese high school students [8], German university students and high school students [9], Japanese university students [10], Polish adults (mostly college students) [11], and Turkish university students [12]. However, the two-factor structure of the Grit-S was not replicated in a Filipino sample [13]. Unfortunately, most of the samples in previous studies were of college students; as such, understanding about the construct of the Grit-S within the broader population is limited. Thus, studies with non-student samples are needed to extend the generalization of the grit instrument.

Previous studies demonstrated that the Grit-S measures grit invariantly across genders in both western [1, 9, 11] and Southeast Asian samples [14]. The measurement invariance (MI) enables the exclusion of any measurement artifact in cross-group comparisons such those between males and females. The MI needs to be satisfied before making comparisons across groups using the scores of the scales [15, 16]. In a Chinese context, it is unclear whether the Grit-S is measuring the same construct in males and females.

With regards to the criterion validity, extensive studies have shown that grit has positive relationships with constructs including but not limited to self-efficacy [17,18], conscientiousness [4, 14, 19, 20], life satisfaction [17,21], and self-control [8, 22]. Meanwhile, grit has negative relationships with depression [18, 23] and stress [24, 25]. It has also been demonstrated that grit can be used to predict empirical or life outcomes. It is well-documented that grittier individuals attain higher levels of education [17], and express higher levels of workplace retention [3], less counterproductive work behaviors [18], and a better working performance [19, 25–27]. The recent meta-analysis of 73 studies (*N* = 66,807) [5] shows that the Grit-S is moderately correlated with both performance and retention. The perseverance of effort factor exhibits the strongest prediction on academic success. The authors recommended examining whether these findings hold true in different domains–such as the workplace–that involve a greater number and range of tasks with different levels of difficulty. To our knowledge, the criteria of empirical validity has not been extended to examine the insurance sector, where professional performance–including status of insurance premiums and commission charges– may contribute to mirror the relationship between grit and performance more explicitly.

### Current Study

The present study aimed to examine the psychometric properties of Grit-S in a large sample of Chinese insurance agency employees. First, it examined the factor structure of the Grit-S by using EFA and CFA. We used half of the sample for an EFA to explore the structure of the Grit-S, then conducted CFA on the other half to examine the factor structure of the model generated from the EFA. We expected that the original two-factor model proposed by Duckworth and colleagues (2007) [1] would fit our sample well [8]. We then tested the MI of the Grit-S across gender and age groups.

Second, we examined the external validity of the Grit-S accounting for more relevant variables, particularly work-related constructs including psychological distress, burnout syndrome, as well as conflict between work and family. Previous studies revealed that psychological distress [28], burnout syndrome [29], and conflict between work and family [30] are all unfavorable situations in which individuals can achieve success, thus, we hypothesized that they are significantly negatively correlated with the total score on the Grit-S. In addition, we analyzed the relationship between the Grit-S scores and the professional performances of the participants (i.e., status of insurance premiums and commission amounts). The hypothesis was that an individual with high-level job preferences would achieve high Grit-S scores.

## Materials and methods

### Participants and procedure

The participants were 2,363 insurance agency employees recruited from 39 insurance companies in Guangdong, China. The age of the respondents ranged from 19 to 70, and approximately 69% were less than 40 years old (mean age = 35.14, SD = 8.99). The descriptive statistics of the participants are summarized in Table 1.

**Table 1:** Demographic summary of participants by different groups (N = 2,363).

Categories	Groups	N	Percentage (%)	Mean of Grit	SD
Age	19 (Lowest) - 29	710	30.0	29.42	5.23
	30–39	866	36.7	29.79	5.13
	40–70 (Highest)	709	30.0	30.12	5.10
	Missing	78	3.33		
Gender	Male	865	36.6	30.02	5.33
	Female	1481	62.7	29.64	5.08
	Missing	17	0.7		
Participant Insurance Premium Status	0–50%	917	38.8	28.95	5.06
	50–80%	539	22.8	29.61	5.02
	80–100%	353	14.9	30.78	5.46
	Above 100%	293	12.4	31.36	5.17
	Missing	261	11.0		
Participant Commission Charges	0–10,000	719	30.4	29.24	5.07
	10,000–50,000	535	22.6	29.40	5.17
	50,000–100,000	505	21.4	29.88	5.15
	Above 100,000	395	16.77	31.12	5.28
	Missing	209	8.8		

All insurance agency employees were administrated at the same time by their companies during morning conferences. Participants took approximately 30 minutes to complete the questionnaire, following a standard procedure. Before participation, participants were informed about the rules and goals of the study, and told they could withdraw from the study at any time. All questionnaire items were written in Chinese. This study was approved by the Human Subjects Review Committee at Guangzhou University.

### Measures

#### Grit-S

The Grit-S is a short-form scale of the GRIT scale, which was developed by Duckworth and Quinn (2009) [4]. The Chinese version was translated by the laboratory of Duckworth (http://angeladuckworth.com/research/). Half of the Grit-S was made up of positively-worded items (e.g., “Setbacks don’t discourage me.”), and half were negatively-worded items (e.g., “I often set a goal but later choose to pursue a different one.”). Respondents selected the options most suitable for themselves on a scale of 1 (“Not at all like me”) to 5 (“Very much like me”).

#### BSI

The BSI (BSI-18, [31]) is a scale that measures the psychological distress that individuals have experienced in the previous seven days. This five-point Likert-like scale measures the severity of the physical and mental state of a person, from 0 (“Not at all”) to 4 (“Extremely”). It has been shown to have satisfactory internal consistency test–retest reliability [32]. The BSI-18 has four factors: somatization, depression, panic, and general anxiety; the alpha coefficients of the current sample for were .87, .86, .88, and .83, respectively.

#### C–MBI

The C–MBI was developed by Maslach and Jackson (1981) [33] to assess individual burnout syndrome via three components: emotional exhaustion, depersonalization, and lack of personal accomplishment [33–35]. Each item was scored on a seven-point scale, from 0 (“The feeling has never been experienced”.) to 6 (“The feeling is experienced daily.”). The C–MBI has good reliability in previous studies [36]. The alpha coefficients of the present study for emotional exhaustion, depersonalization, and lack of personal accomplishment were .81, .72, and .73, respectively, and these values indicated acceptable to good internal consistency.

#### Work–family and family–work conflict scales

The work–family and family–work conflict scale is a five-point Likert-like scale with 12 items representing two factors: work interfering with family, and family interfering with work. Each factor has three dimensions: psychological resource, emotional, and behavioral conflicts [37]. Participants rate options according to the frequency they experience the described situation (1 = “Occurs fairly rarely”; 5 = “occurs always”). In the current study, the alpha coefficients for “work interferes with family” and “family interferes with work” were .82 and .86, respectively.

### Data analysis strategy

First, descriptive statistics of each Grit-S item were calculated. To determine the factor structure of grit, EFA was conducted on a random split-half sample and CFA was applied to the other sample half to find and confirm the optimal model to explain the data. The whole sample was used to test the MI across gender and age groups. Next, the group differences were examined by comparing the means of the overall grit scores. Finally, zero-order correlation was used to compute criterion validity, and the differences in grit score among the insurance employees were tested through one-way ANOVA. The descriptive statistics were calculated by SPSS (IBM, SPSS Version 19, 2010). EFA and CFA were conducted by Mplus 8.0 [38].

#### Stage 1: Factor structure

Robust weighted least squares with mean and variance adjustment (WLSMV) was a suitable method for the estimator because the data was categorical [39–41]; thus, EFA with the WLSMV estimator with oblique rotation was conducted to identify the factor structure of the Grit-S. Additionally, parallel analysis and a scree plot were used with the robust maximum likelihood estimator to determine the number of factors [42].

To confirm the structure generated from EFA, CFA was conducted using the WLSMV estimator. Fit indices, such as chi-squares, root mean square error of approximation (RMSEA), Tucker–Lewis index (TLI), and comparative fit index (CFI), were obtained to evaluate the goodness-of-fit of the model. An RMSEA value smaller than .08 represents an acceptable fit, whereas a value of .06 or lower indicates good fit [43, 44]. TLI and CFI values higher than .90 indicate an acceptable fit, and values exceeding .95 represent an excellent fit [45].

#### Stage 2: MI and mean comparisons

MI tests were conducted across gender and age groups using multi-group CFA. Each group model generated from EFA and examined in CFA was initially assessed before conducting the MI test [46]. Next, four levels of MI tests (i.e., configural, metric, scalar invariance, and error variance) were carried out. The differences in fit indices between the unconstrained and constrained models were obtained to examine whether a specific level of MI was achieved. Because the chi-square test is sensitive to sample size and small differences yield considerable variations if the sample is too large [47, 48], we considered the difference in CFI (ΔCFI) and TLI (ΔTLI) as suitable indicators of MI [49]. According to Cheung and Rensvold (2002) [49], the equivalence is acceptable when ΔCFI ≤ .010 and ΔTLI ≤ .010.

The mean comparisons between gender and age groups were then conducted. If Grit-S is invariant across gender and age groups, then a comparison between different gender and age groups is also valid. The mean difference of gender and age groups was analyzed via *t-*test and one-way ANOVA, respectively, using SPSS (IBM, SPSS Version 19, 2010). The statistical tests adopted a significance level of .05.

#### Stage 3: Criterion and empirical validity

Criterion validity of Grit-S was assessed via correlations calculated by SPSS (IBM, SPSS Version 19, 2010). Empirical validity was examined by one-way ANOVA by comparing the differences in grit scores among participants divided by different levels of completion status of insurance premiums or commission amounts. To examine whether the two factors had correlations with the external criterion in a similar magnitude, Z values (*p* < .001, two-tailed for significance) were calculated on the basis of Dunn and Clark’s (1969) method [50], using a spreadsheet developed by DeCoster and Lselin (2005) which can be retrieved at: http://stat-help.com/spreadsheets.html.

## Results

### Descriptive statistics and factor structure

**Fig 1:**
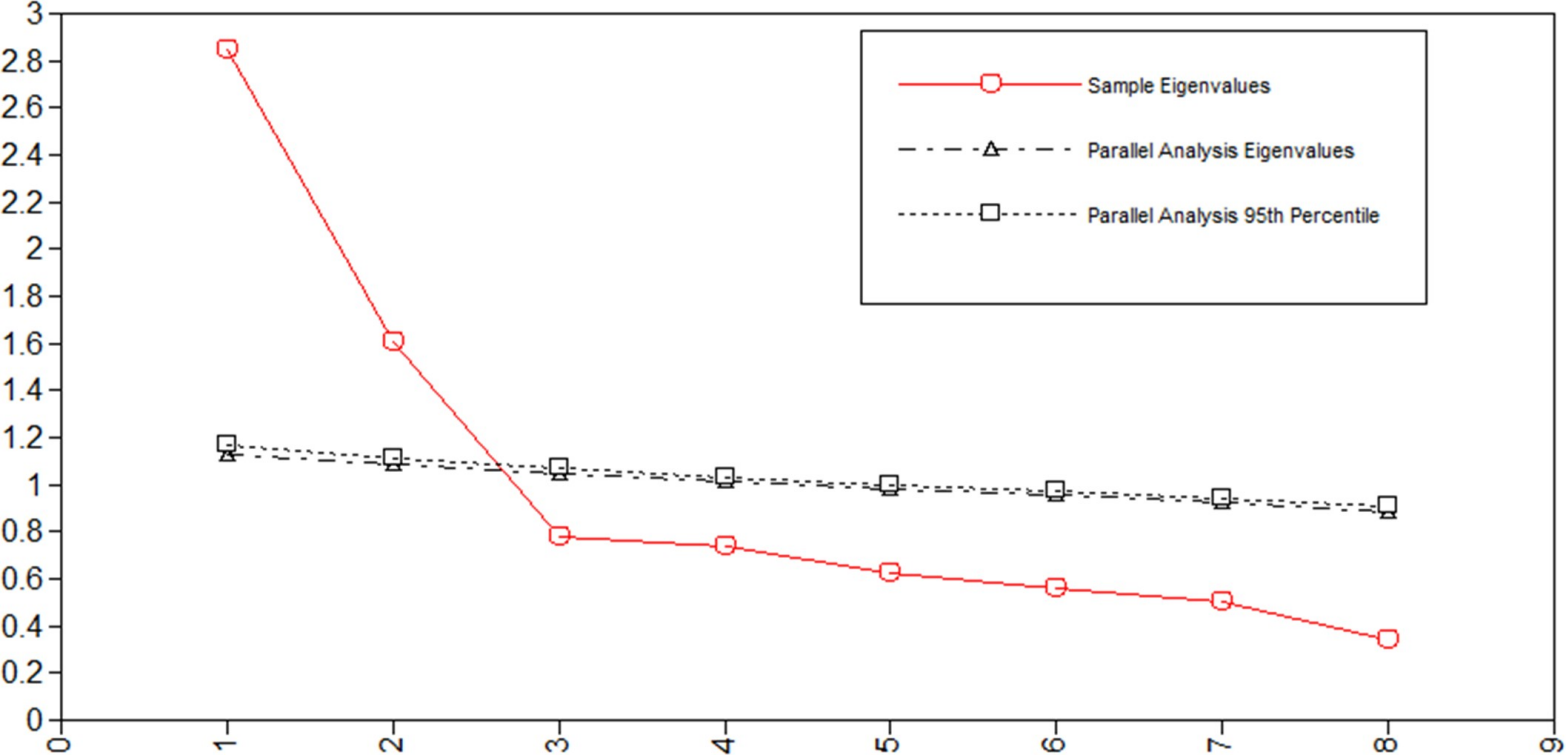
Scree plot for EFA.

The descriptive statistics, including means, standard deviations, skewness and kurtosis, were summarized in Table 2. We used a random split-half sample (*N* = 1,181) to conduct EFA with the WLSMV estimator. This analysis yielded two factors with eigenvalues of more than 1.00 (2.84 and 1.61). Furthermore, the scree plot test and parallel analysis suggested that the two-factor model was suitable. Fig 1 shows the results of the parallel analysis in detail. Each item was loaded onto its target factor, and all factor loadings were significant at *p* < .001. In line with prior studies [4, 10, 12], the current Grit-S could be best explained by a two-factor model using consistency of interest (item: 1, 3, 5, 6) and perseverance of effort (item: 2, 4, 7, 8). The detailed statistics are summarized in Table 2. The correlation between the two factors was modest (*r* = .49, *p* < .001). The reliability analysis indicated that the alpha coefficients for the overall grit score, consistency of interest, and perseverance of effort were .85, .70, and .75, respectively.

**Table 2:** Descriptive statistics of Grit-S and standardized factor loadings of the original two-factor model.

	Descriptive statistics				Factor	
Item	Mean	SD	Skewness	Kurtosis	Interest	Effort
1. New ideas and . . .R	2.492	1.038	.190	-.323	0.463***	
3. I have been …R	2.322	1.090	.432	-.477	0.641***	
5. I often set …R	2.330	1.123	.493	-.517	0.745***	
6. I have difficulty …R	2.296	1.097	.558	-.382	0.693***	
2. Setbacks don’t …	3.471	1.246	-.396	-.800		0.468***
4. I am a hard …	3.998	1.059	-.934	.270		0.733***
7. I finish whatever …	3.833	1.096	-.637	-.384		0.801***
8. I am diligent.	3.982	1.038	-.799	-.058		0.904***

*** *p* < .001; R = reversed score; Interest = consistency of interest; Effort = perseverance of effort.

CFA was performed on another split-half sample (*N* = 1,182), and the results showed an acceptable goodness-of-fit for the two-factor model (WLSMV *χ*^2^ = 102.047, *df* = 19, CFI = .986, TLI = .979, RMSEA = .06).

### MI and mean comparison

MI was performed across gender and age groups. Gender was divided into male (*N* = 865) and female (*N* = 1,481) groups, and age was divided into three groups: Lowest thru 29 (*N* = 709), 30–39 (*N* = 866), and 40 thru Highest (*N* = 705). Additional demographic characteristics are shown in Table 1.

The results of the MI are shown in Table 3, and two fit indices (i.e., CFI, TLI) indicated an acceptable fit of the two-factor model (CFIs > .95, TLIs > .95). Although significant Δ*χ*^*2*^ occurred when examining the invariance, other fit indices did not exceed the threshold of .01; this finding indicated that scale equivalence on gender and age for the four models (configural, metric, scalar, and error variance) was acceptable. Overall, the results of the MI indicated that Grit-S was equivalent across gender and age groups.

**Table 3:** Goodness-of-fit indices and model comparisons for measurement invariance models.

Type	Model	WLSMVχ^2^	*df*	TLI	CFI	RMSEA (90% CI)	Comparison	Δχ^2^	Δ*df*	ΔTLI	ΔCFI
Age	A-Configural invariance	204.068**	57	.979	.986	.058 [.050-.067]					
	B-Metric invariance	218.649**	69	.982	.985	.053 [.046-.061]	B VS.A	28.58*	12	+.003	+.001
	C-scalar invariance	252.749**	113	.990	.986	.040 [.034-.047]	C VS.B	42.116	44	+.008	+.001
	D-Error Variance invariance	356.781**	129	.985	.978	.048[.042-.054]	D VS.C	109.547**	16	-.005	-.008
Gender	A-Configural invariance	193.703**	38	.978	.985	.059 [.051-.068]					
	B-Metric invariance	164.902**	44	.983	.986	.052 [.045-.060]	B VS.A	7.815	6	+.005	+.001
	C-scalar invariance	222.347**	66	.987	.985	.045 [.039-.052]	C VS.B	44.466**	22	+.004	-.001
	D-Error Variance invariance	230.847**	74	.989	.985	.043 [.036-.049]	D VS.C	12.941	8	+.002	.000

WLSMV = weighted least squares with mean and variance adjustment; *df* = degree of freedom; TLI = Tucker-Lewis index; CFI = comparative fit index; RMSEA = root mean square error of approximation; CI = confidence interval; Δχ 2 = change in χ 2 relative to the preceding model; Δ*df* = change in degree of freedom relative to the preceding model; ΔCFI = change in comparative fit index relative to the preceding model; ΔTLI = change in Tucker-Lewis index relative to the preceding model. Chi-square difference test with WLSMV estimation is different from the conventional chi-square difference test; more information on this can be found at: http://www.statmodel.com/chidiff.shtml.

*p < .05.

**p < .01.

Results from the *t*-test and one-way ANOVA indicated that the mean grit score of male sample groups was not significantly different from that of the female groups, *t*_(0.05/2, 2344)_ = 1.74, *p* > .05, *d* = .07. However, the mean grit score was significantly different across different age groups: *F*_(2, 2285)_ = 3.34, *p* < .05,*η*^*2*^ = .003. The post-hoc test suggested that only the difference between the age groups of below-29 and above-40 was significant (mean difference = -.706, *p* < .05).

### Criteria and empirical validity

Results of the zero-order correlation analysis showed that the Grit-S had acceptable criterion validity in this investigation. The two-factor Grit-S total scores were significantly negatively correlated with BSI, C–MBI, and work–family and family–work conflict scores. The details of correlation and descriptive statistics among these variables are presented in Table 4.

**Table 4:** Means, standard deviations, and the intercorrelation matrix among study variables.

	Variable	Mean	SD	1	2	3	4	5	6	7	8	9	10	11	12	13	14	15	16	17
1	Interest	14.486	3.130	1																
2	Effort	15.284	3.339	.280**	1															
3	Grit-S	29.770	5.178	.785**	.814**	1														
4	C-MBI-E	16.841	6.895	-.372**	-.174**	-.337**	1													
5	C-MBI-D	10.392	4.706	-.364**	-.300**	-.413**	.476**	1												
6	C-MBI-L	13.517	5.041	-.260**	-.489**	-.473**	.170**	.298**	1											
7	C-MBI-T	40.750	12.326	-.453**	-.412**	-.540**	.811**	.770**	.618**	1										
8	BSI-S	8.112	3.484	-.196**	-.120**	-.196**	.426**	.334**	.103**	.408**	1									
9	BSI-D	9.137	4.135	-.322**	-.223**	-.339**	.546**	.384**	.203**	.535**	.713**	1								
10	BSI-A	5.068	2.497	-.274**	-.168**	-.274**	.541**	.330**	.146**	.489**	.714**	.825**	1							
11	BSI-P	4.196	2.264	-.259**	-.165**	-.263**	.448**	.311**	.124**	.420**	.713**	.788**	.773**	1						
12	BSI-T	26.512	11.196	-.293**	-.191**	-.300**	.546**	.382**	.165**	.519**	.878**	.935**	.906**	.888**	1					
13	WIF	11.495	4.453	-.243**	-.089**	-.204**	.496**	.300**	.093**	.430**	.492**	.540**	.529**	.487**	.569**	1				
14	FIW	10.529	4.240	-.274**	-.160**	-.269**	.437**	.335**	.146**	.432**	.474**	.530**	.515**	.482**	.555**	.760**	1			
15	Gender	1.631	.483	-.029	-.028	-.036	.010	-.103**	.030	-.021	.007	-.052*	-.018	-.021	-.025	-.017	-.049*	1		
16	Age	35.143	8.985	.051*	.031	.051*	.016	.025	-.054*	-.003	-.006	-.058**	-.063**	-.047*	-.047*	.049*	.030	.172**	1	
17	Premium	2.010	1.078	.166**	.113**	.173**	-.022	-.073**	-.206**	-.125**	.010	-.047*	-.033	-.030	-.028	.087**	-.010	-.037	.150**	1
18	Charges	2.303	1.179	.100**	.094**	.121**	.024	.011	-.181**	-.057**	.073**	.010	.034	.032	.041	.129**	.043*	.011	.162**	.623**

* *p* < .05

** *p* < .01

Interest = consistency of interest; Effort = perseverance of effort; C-MBI-E = C-MBI-Emotional Exhaustion; C-MBI-D = C-MBI- Depersonalization; C-MBI-L = C-MBI-Lack of Personal Accomplishment; C-MBI-T = C-MBI-Total; BSI-S = BSI-Somatization; BSI-D = BSI-Depression; BSI-G = BSI-General Anxiety; BSI-P = BSI-Panic; BSI-T = BSI-Total; WIF = work interferes with family; FIW = family interferes with work; Premium = participant status in insurance premiums; Charges = participant commission charges.

**Fig 2:**
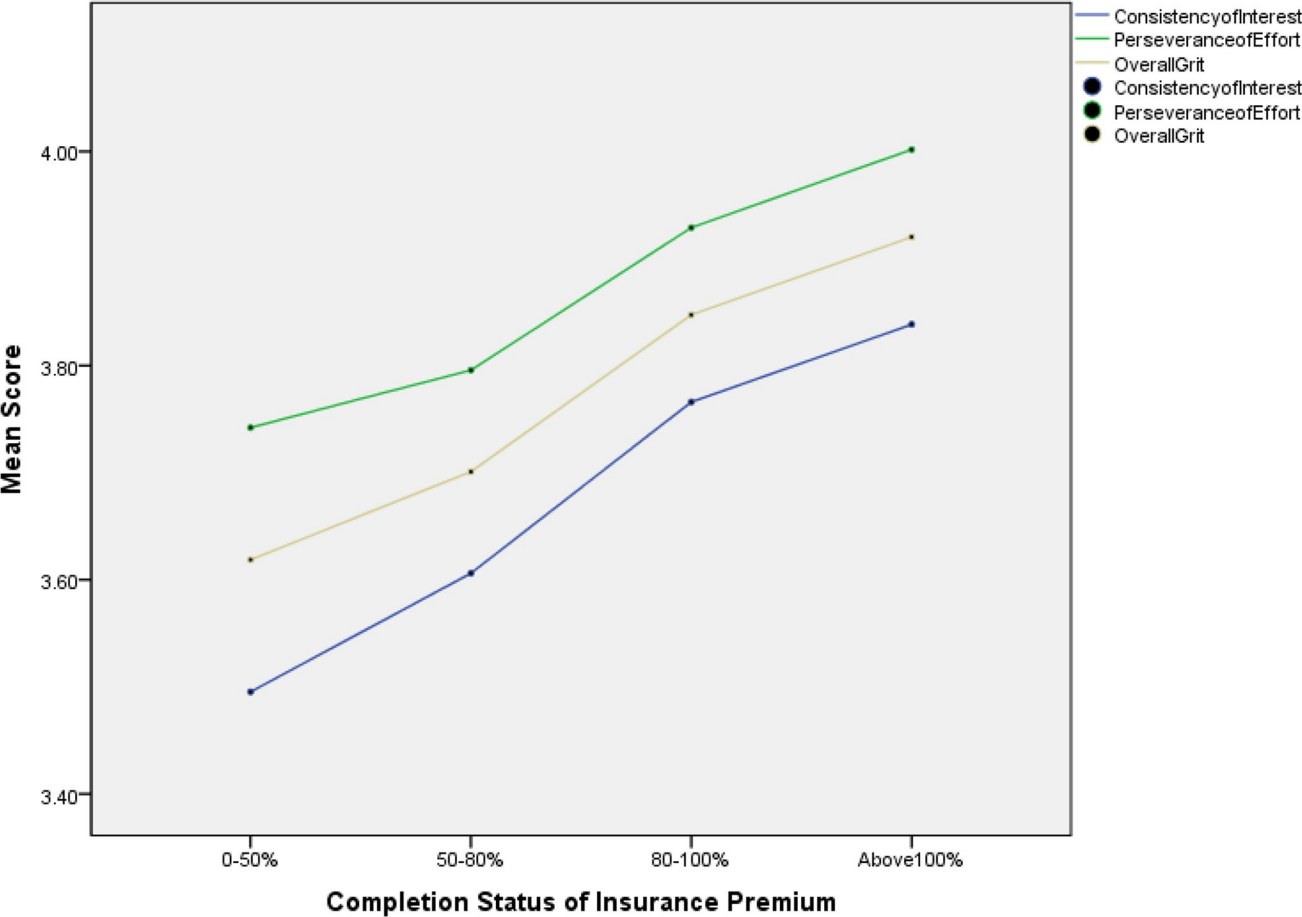
Mean plot of insurance employees’ Grit-S scores on different statuses of insurance premiums.

**Fig 3:**
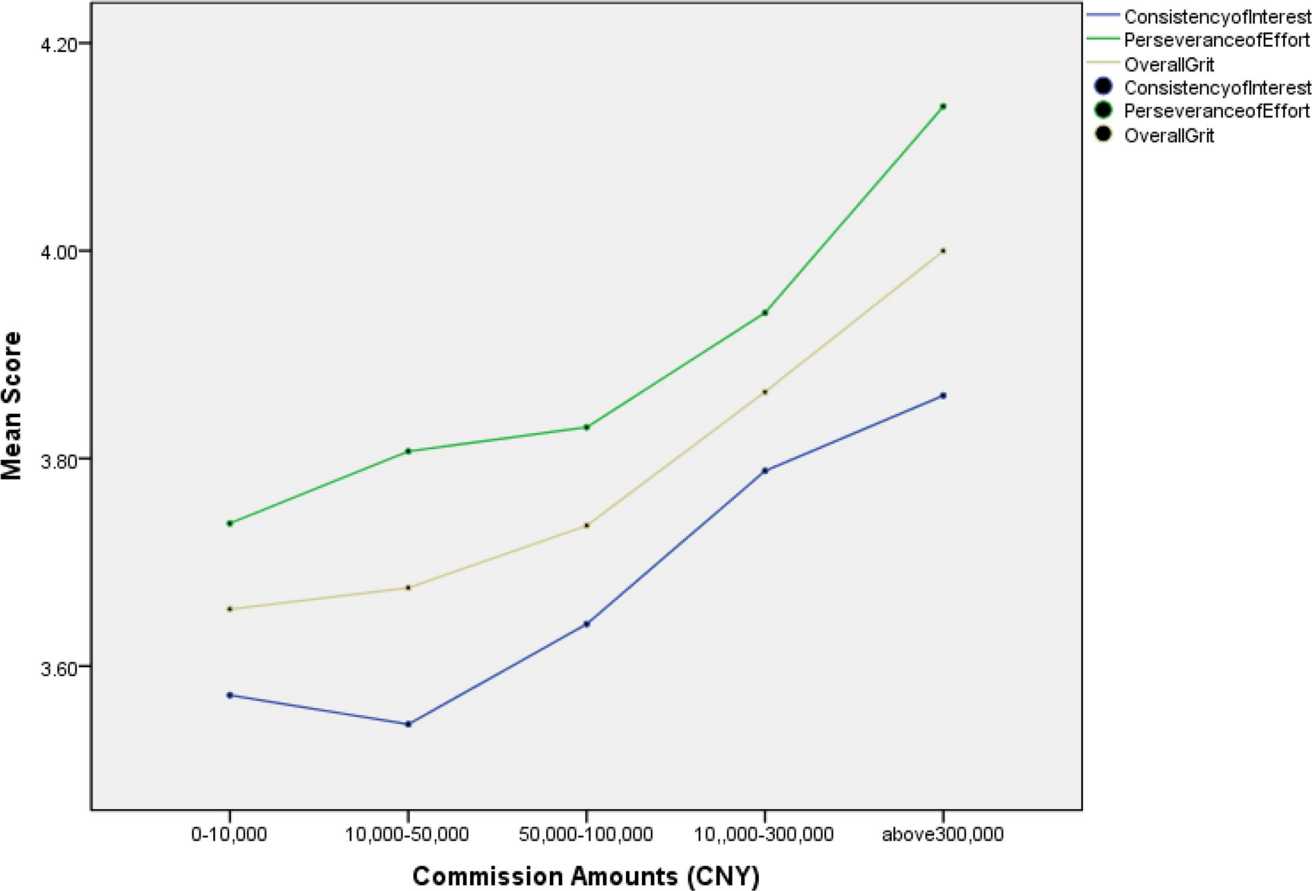
Mean plot of insurance employees’ Grit-S scores on different levels of commission amounts.

Furthermore, insurance employees with different levels of job performance showed different level scores on the Grit-S. The higher scores on the Grit-S correlated with higher group status levels in insurance premiums (*F*_(3,2359)_ = 21.92, *p* < .001,*η*^*2*^ = .03) and commission amounts (*F*_(5,2357)_ = 8.16, *p* < .001,*η*^*2*^ = .02). These results are also depicted in Figs 2 and 3.

**Fig 4:**
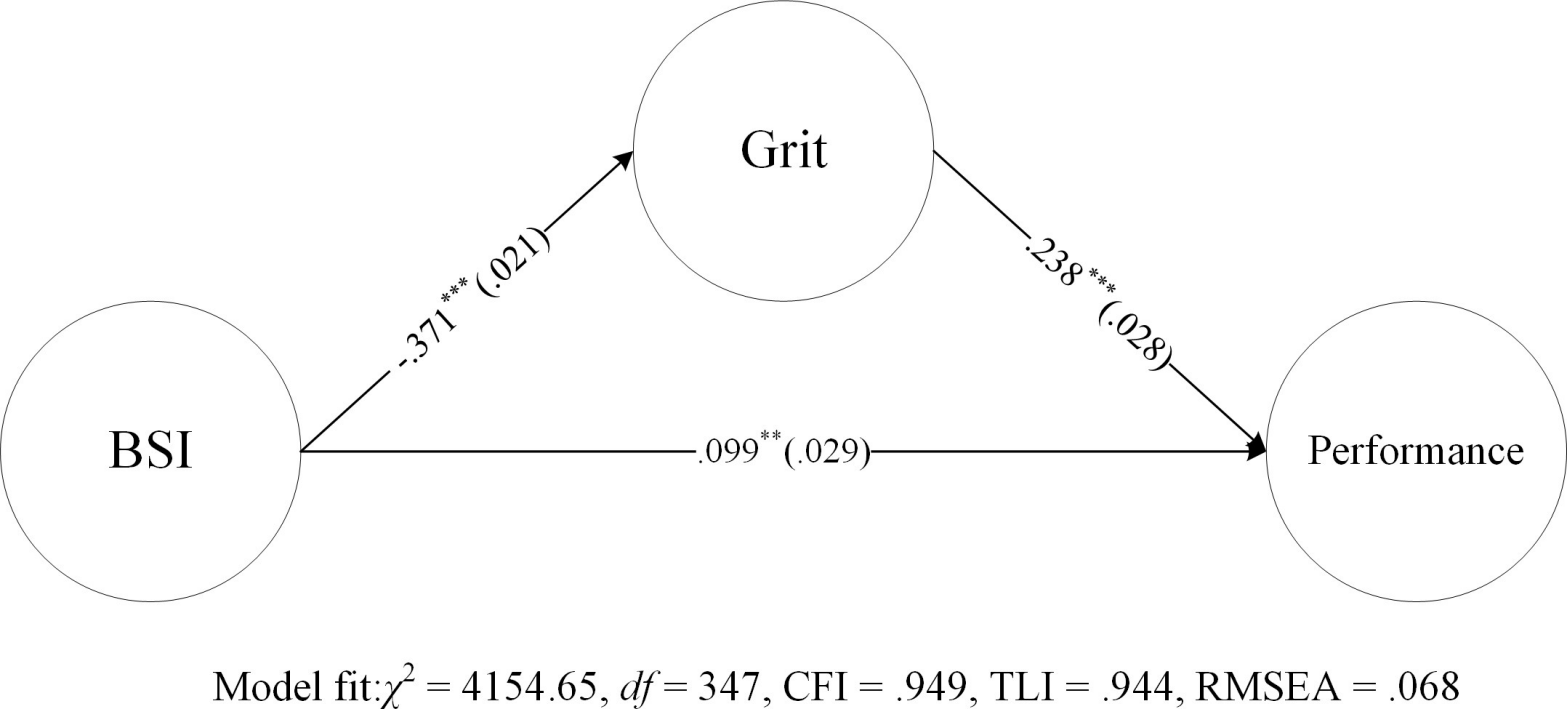
Mediation model and significant simple mediation results. Standardized coefficients (standard errors), ** *p* < 0.01, *** *p* < 0.001.

To further examine how well grit and psychological wellbeing (BSI) can predict job performance, mediation analysis with the WLSMV estimator was carried out. A significant mediation pathway was found between the BSI and job performance through the Grit-S (see Fig 4). The indirect effect of BSI on job performance is—.088, *p* < .001, and the ratio of intermediary effect to total effect is 47.06%, suggesting that the Grit-S substantially mediated the effect of BSI on job performance.

To compare the different functions of the two facets of grit, the difference in the magnitudes of their correlations with external variables were tested. As shown in Tables 4 and 5, the consistency of interest and perseverance of effort had significantly different correlations with all criteria variables except for BSI and commission amounts. The consistency of interest factor had stronger relationships with those variables than the perseverance of effort did.

**Table 5:** Summary of comparing two dependent correlations.

	Z	*p*-value (two-tailed)
BSI	1.927	0.054
C-MBI	4.351	0.000
Work-family conflict	6.417	0.000
Family-work conflict	4.791	0.000
Completion status of insurance premiums	2.174	0.030
Commission amounts	0.238	0.812

## Discussion

This study aimed to examine the psychometric properties and MI of the Grit-S. EFA and CFA indicated that the original two-factor model of the Grit-S fit the data well. In addition, the Grit-S was supported by the MI, as the means difference was not significant across gender, but was significant with a small effect size across age groups. The overall two-factor (i.e., perseverance of effort and consistency of interest) Grit-S exhibited significant correlations with external criteria. Furthermore, empirical validity was demonstrated by the significant relation between participant grit and job performance of participants.

The first aim of this work was to test the factor structure of the Grit-S in mainland Chinese adults. The results of EFA and CFA supported our hypothesis that the original two-factor structure fit our sample well. The structure also demonstrated MI across genders and age groups. These findings provide evidence for the applicability of the Grit-S in Chinese professional samples.

Next, we found that there were no significant differences between the scores of the two genders on the Grit-S. This is in line with previous findings in western samples [4, 10, 12]. However, significant score differences were found between the 19–29 age group and the 40–70 age group. A previous study had proposed that grit increases significantly with age [1], and our findings are partly consistent with this previous study, but the effect size is small. One possible reason for this is that we endorsed an age group classification (19–29, 30–39, and 40–70) different from that used in the previous study (25–34, 35–44, 45–54, 55–64, and 65 and above). Hence, we suggest that further investigations adopt older participants than those used in this study when comparing grit across different age levels.

The Grit-S showed criteria and empirical validity when used for mainland Chinese participants. In the current study, we selected variables more specifically related to work, including psychological distress, burnout syndrome, and conflicts between work and family. The Grit-S was significantly negatively correlated with these external variables. The negative correlations between grit and these psychopathological variables further examined the proposition that grit is a significant indicator of success and performance [1–3]. Insurance agents not only need to have a good knowledge about insurance offerings and regulations, but also must form relationships and build trust with customers. This process of relationship building requires both endurance and the ability to cope with rejection from customers, which are essential features of grit.

Grit was correlated significantly with psychological wellbeing (BSI), indicating that individuals with high Grit-S scores were inclined to experience less somatization, depression, panic, anxiety, burnout syndrome, and work–family conflicts than those with low scores. We explored how grit and BSI predict job performance via mediation analysis and found that grit significantly explained the contribution of psychopathological factors (BSI) on job performance. This suggests that the negative impact of poor psychological wellbeing on job performance can be partially incurred by a lack of or decrease in grit.

Finally, we found that the consistency of interest factor showed significantly a stronger correlation with the criteria than perseverance of effort did, except for with BSI and participants' commission amounts. This result is not in line with previous findings, that perseverance of effort showed a stronger relationship with performance [5, 13]. Consistency of interest is promoted widely in mainland China in colloquial forms, such as in sayings like, for example, “three days fishing, two days drying nets” or “constant dripping will wear away a stone.” Therefore, mainland Chinese might think highly of consistency of interest when compared to people living in other nations. With regards to commission amounts, the complexity of the insurance occupation may demand the two factors (i.e., BSI and commission amounts) work together. However, the underlying functional mechanism of grit and its relation to professional criteria and performance has not been explored thoroughly yet. This therefore calls for the need to carry out mechanism investigations considering more contributing factors.

Some limitations existed in this current study. We selected a sample of Chinese insurance employees from around the same region of the country. Consequently, the findings may not be generalized to individuals in other areas of mainland China. Furthermore, the current study used only a Chinese sample, without the inclusion of samples from other cultural contexts. Thus, there is a lack of direct cultural comparison. It is unclear if the Grit-S measures grit in the same way across different cultures. Similarly, the profile of our sample allowed us to explore grit in a single professional domain. However, the use of only one profession may limit the generalizability of the results to individuals from other occupations. Therefore, further studies should extend investigations to different occupations. Another limitation is that all the items measuring consistency of interest are negatively-worded, and items measuring perseverance of effort are positively-worded, which also may have influenced the EFA and CFA in exploring the structure of grit [51]. A third limitation is the reliance on self-reported measures for the criteria and empirical validity analyses. Such a methodology introduces shared method variance, which can inflate the magnitude of observed correlations. The cross-sectional design of our research is another methodological limitation that constrained observations of the development of external variables, such as BSI, C–MBI, the work–family and family–work conflict scales, as well as the performances of participants; therefore, a longitudinal design in future studies would be beneficial.

In summary, our findings suggest that the Grit-S is a reliable and effective instrument when using with two factors (consistency of interest and perseverance of effort) for measuring grit in Chinese employees. The current study provides sound support for the MI in comparing gender and age group differences using the Grit-S. Furthermore, the Grit-S plays an important role in measuring people’s psychological factors and performance.

## Supporting information

S1 File Datasets-grit in chinese. (XLSX)
